# Patellar Non-Traumatic Pathologies: A Pictorial Review of Radiologic Findings

**DOI:** 10.3390/diagnostics14242828

**Published:** 2024-12-16

**Authors:** Zahra Masroori, Sara Haseli, Elahe Abbaspour, Alireza Pouramini, Arash Azhideh, Marjan Fathi, Fatemeh Kafi, Majid Chalian

**Affiliations:** 1 Department of Radiology, Division of Musculoskeletal Imaging and Intervention, University of Washington, Seattle, WA 98105, USA; 2 OncoRad Research Core, Department of Radiology, University of Washington/Fred Hutchinson Cancer Center, Seattle, WA 98195, USA

**Keywords:** non-traumatic patellar pathologies, patellofemoral imaging, radiology, patellar tendinopathy, chondromalacia, patellar dysplasia

## Abstract

Patellar pathologies are a common cause of knee dysfunction, with Patellofemoral Pain Syndrome (PFPS) alone responsible for 25% of knee-related visits to sports medicine clinics. Non-traumatic conditions, while often overlooked, can also lead to significant discomfort and functional limitations, highlighting the importance of accurate and timely diagnosis for effective management and prevention of complications. This pictorial review examines the radiologic characteristics of various non-traumatic patellar disorders, focusing on imaging modalities such as radiography, computed tomography (CT), and magnetic resonance imaging (MRI). Key diagnostic markers, including patellar tilt, tibial tuberosity–trochlear groove distance (TT-TG), and congruence angle (CA), are discussed for their significance in non-traumatic pathology identification. Furthermore, this review highlights specific radiologic features for a range of non-traumatic patellar conditions, including patellar tendinopathy, chondromalacia patellae, and trochlear dysplasia, emphasizing how distinct radiologic findings facilitate precise diagnosis and clinical assessment. Ultimately, it provides a practical guide for clinicians in diagnosing non-traumatic patellar pathologies through a comprehensive review of key radiologic features while also discussing advancements in imaging technologies and management strategies to support accurate diagnosis and effective clinical decision-making.

## 1. Introduction

Knee pain accounts for approximately 5% of all adult primary care visits [1]. One of the most common underlying causes of knee pain is Patellofemoral Pain Syndrome (PFPS), which is reported in about 25% of knee injuries observed in sports medicine clinics [2]. The patella connects the quadriceps tendon to the patellofemoral ligament. Protecting the deeper structures of the knee joint, it helps in directing the forces generated by the quadriceps femoris muscle toward the patellar ligament and reduces the frictional damage to the quadriceps tendon [3,4].

**Figure 1:**
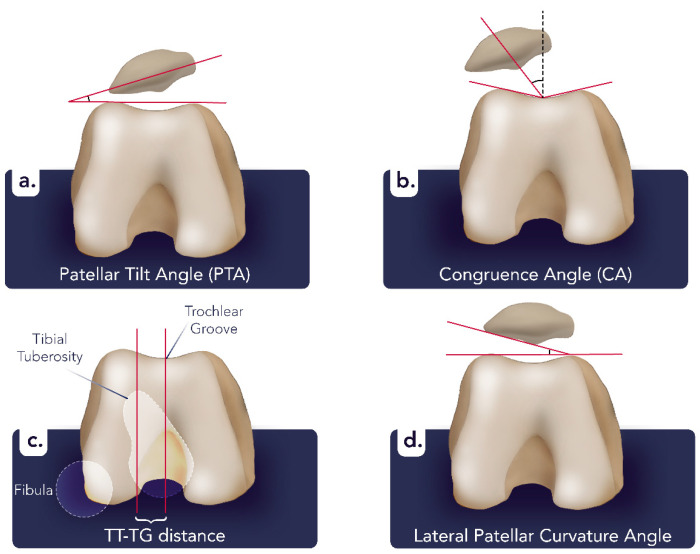
Merchant view of the patellofemoral joint demonstrating key anatomical angles and measurements. Patellar tilt angle (PTA) (**a**): the angle formed between a line connecting the anterior points of the medial and lateral facets of the patella and a line tangent to the femoral condyles. Normally, it is less than 5° laterally. An increased patellar tilt angle can lead to patellar maltracking. Congruence angle (CA) (**b**): the angle between a line drawn perpendicularly to the deepest point of the trochlear groove and a line from the same point to the apex of the patella. Normally, it is −6° (within a range from −6° to +16°). A positive CA suggests lateral displacement of the patella, which may be associated with patellar subluxation or lateral tracking issues. Tibial tubercle–trochlear groove (TT-TG) distance (**c**): the horizontal distance between the lowest point of the trochlear groove and a line connecting the highest points of the medial and lateral femoral condyles, best measured on CT. Generally, a TT-TG distance under 15–20 mm is considered normal. A measurement exceeding this range suggests a lateralized tibial tubercle, which can affect patellar tracking. Lateral patellar curvature angle (LPCA) (**d**): the angle is formed between a line along the lateral facet of the patella and a perpendicular line to the patella’s axis. While normal values vary, a sharper lateral patellar curvature angle indicates a steeper lateral facet, increasing contact pressures on the lateral patellofemoral joint and contributing to lateral patellar tilt or maltracking. The image was obtained from the University of Washington Medical Imaging database with the required permissions.

There are several radiographic techniques for the evaluation of patellar and patellofemoral joint pathologies, including lateral, anteroposterior (AP), and Merchant (notch) projections on conventional radiography. The AP view can detect varus and valgus misalignment, medial or lateral compartment constriction, and arthrosis. The lateral view assesses patellar height, dislocations, fractures, osteoarthritis, and the functional relationship between the patella and the tibia, as well as the patellar facets and the femur. The Merchant view is useful for evaluating osteochondral fragments, patellofemoral joint space reduction, the sulcus angle, and the congruence angle (CA) and for assessing trochlear groove depth and patellar tilt [4,5] (Figure 1). While computed tomography (CT) imaging at different flexion angles is useful in the evaluation of osseous anatomy, magnetic resonance imaging (MRI) is particularly effective for evaluating soft tissue characteristics, including the patellofemoral retinaculum, trochlea, quadriceps tendon, patellar ligament, and articular cartilage. Fluid-sensitive T2-weighted images are more sensitive to providing greater contrast between the cartilage surface and joint compared to T1-weighted images [6,7,8]. In this pictorial review, we have highlighted the radiological characteristics of the most prevalent non-traumatic patellar disorders.

## 2. Anatomical Variants

### 2.1. Bipartite/Multipartite Patella

**Figure 2:**
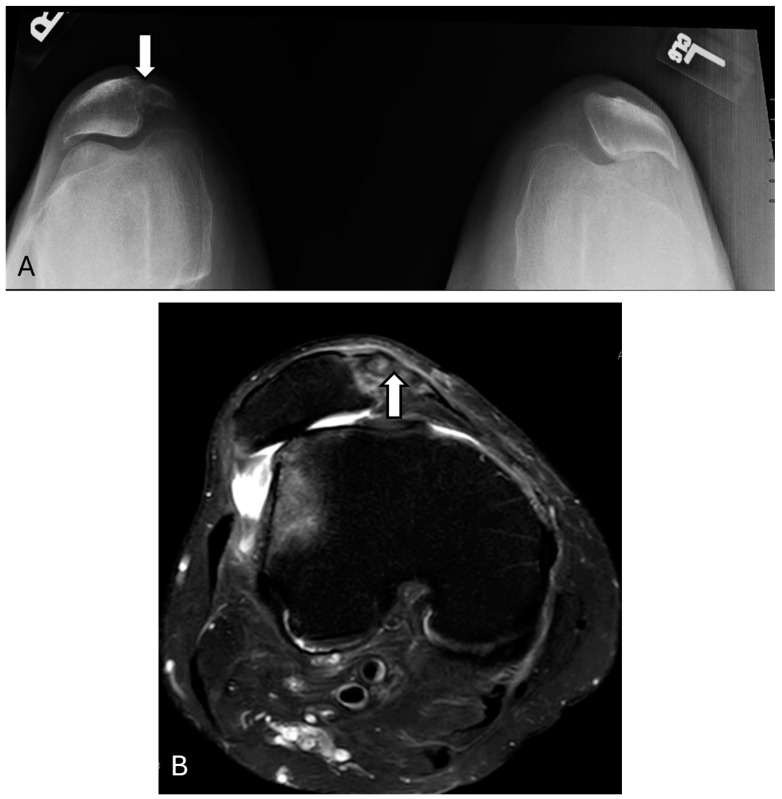
Bipartite patella. The sunrise view knee X-ray (**A**) shows a right-sided bipartite patella (arrow). Lateral subluxation of the patella, more prominent on the left side, is likely degenerative. Axial proton density image (**B**) confirms a shallow trochlea with an edematous bipartite patella (arrow) and marrow edema within the lateral femoral condyle. The images were obtained from the University of Washington Medical Imaging database with the required permissions.

**Figure 3:**
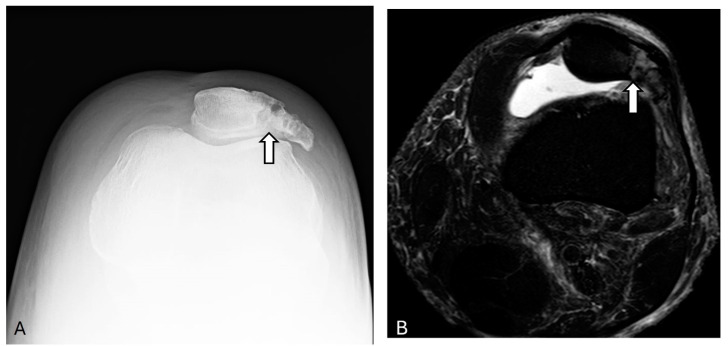
Multipartite patella. Knee X-ray (**A**) and axial proton density fat-suppressed image (**B**) reveal multipartite patella (arrows) with lateral irregularities and edema in the bone and lateral retinaculum suspicious for abnormal motion between the patellar and bipartite portion. The images were obtained from the University of Washington Medical Imaging database with the required permissions.

Bipartite or multipartite patella occurs as a failure of the fusion of primary and secondary ossification centers of the patella during adolescence. Its incidence is estimated to be between 2% and 6%, with a higher prevalence among younger men [9,10]. It is often an incidental finding, and patients are asymptomatic. However, its differentiation from patellar fracture could be challenging. If the fragmentation extends beyond the upper and lower outer quadrants of the patella, a traumatic fracture should be suspected. Radiography usually shows a smooth, well-corticated border around the fragmented patella without any evidence of osteophyte growth [11]. (Figure 2 and Figure 3) Symptoms, if present, usually manifest after strenuous physical activity involving repeated knee extension. Additionally, a weight-bearing skyline view can reveal the separation of the accessory fragment, which may indicate a symptomatic bipartite patella. Additionally, MRI may show adjacent bone marrow edema associated with the accessory fragment [12,13].

### 2.2. Dorsal Defect of the Patella

**Figure 4:**
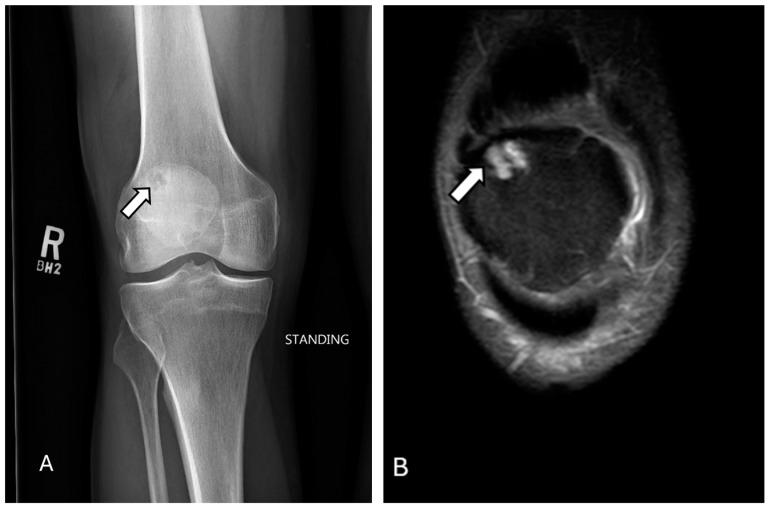
Dorsal defect of patella. Anterior–posterior knee X-ray (**A**) and coronal proton density fat-suppressed image (**B**) show a well-marginated 12 mm lesion (arrows) at the superolateral aspect of the dorsal patella with no associated marrow edema, most likely representing a dorsal patellar defect. The images were obtained from the University of Washington Medical Imaging database with the required permissions.

The dorsal defect of the patella is a benign, circular radiolucent lesion with a sclerotic border typically found in the superolateral aspect of the patella near the subchondral bone (Figure 4). This anatomical variation is asymptomatic in approximately 75% of cases. On T2-weighted MR images, the defect is clearly visible, while on T1-weighted MR images, it appears as a low signal area [14,15].

Certain features can help differentiate dorsal patellar deficits from osteochondral lesions, chondroblastoma, and osteochondritis dissecans. Osteochondritis dissecans are characterized by their location on the convexity of the patella, with a tendency for the medial rather than the lateral facet. In addition, it involves both the articular cartilage and subchondral bone and often presents with a bony fragment [16,17]. In contrast, chondroblastomas are slow-growing, non-resolving, and painful lesions that appear osteolytic, spherical, or lobulated on radiographs, with a well-defined sclerotic rim of reactive bone [18].

## 3. Pathologic Conditions of Patella

### 3.1. Acute Patellar Subluxation and Dislocation

Dislocation and intermittent subluxations represent the two forms of patellar instability. Acute non-traumatic patellar dislocations are commonly caused by congenital conditions, such as Down syndrome, generalized ligamentous laxity, or muscular imbalance (particularly, weakness of the vastus medialis obliquus muscle). These cases frequently present with joint effusion or hemarthrosis and are associated with acute anterior or anteromedial knee pain that worsens with activities such as bending or kneeling [19]. For further evaluation, radiographs of the injured knee are taken in multiple views (e.g., Merchant, lateral, and AP). CT scans are particularly useful for assessing osteochondral fractures and planning surgical interventions. In contrast, MRI remains the preferred imaging technique for evaluating soft tissue injuries, including damage to the medial patellofemoral ligament and cartilage defects [19,20,21,22].

To examine potential subluxation, bend the knee to a 20-degree angle and use the lateral patellofemoral angle, formed by a line that connects the medial and lateral trochlear facets and tangential to the patella’s lateral facet, which typically opens laterally. Another option is to use the patellofemoral index, which is determined as the ratio of medial to lateral joint space thickness, with a normal value of 1.6 inches or less [23].

### 3.2. Chronic Patellar Instability and Malalignment

Chronic patellar instability occurs due to an imbalance in patella–trochlea dynamics as a result of an underlying anatomical or morphological defect, such as excessive ligamentous laxity [24]. The most common presentation is recurrent lateral patellar instability, rarely accompanied by anterior knee pain [25,26,27]. Women are particularly predisposed to patellar malalignment due to anatomical factors involving the patella, tibia, and pelvis, as well as postural habits, such as sitting with legs adducted and wearing high heels [28,29].

**Figure 5:**
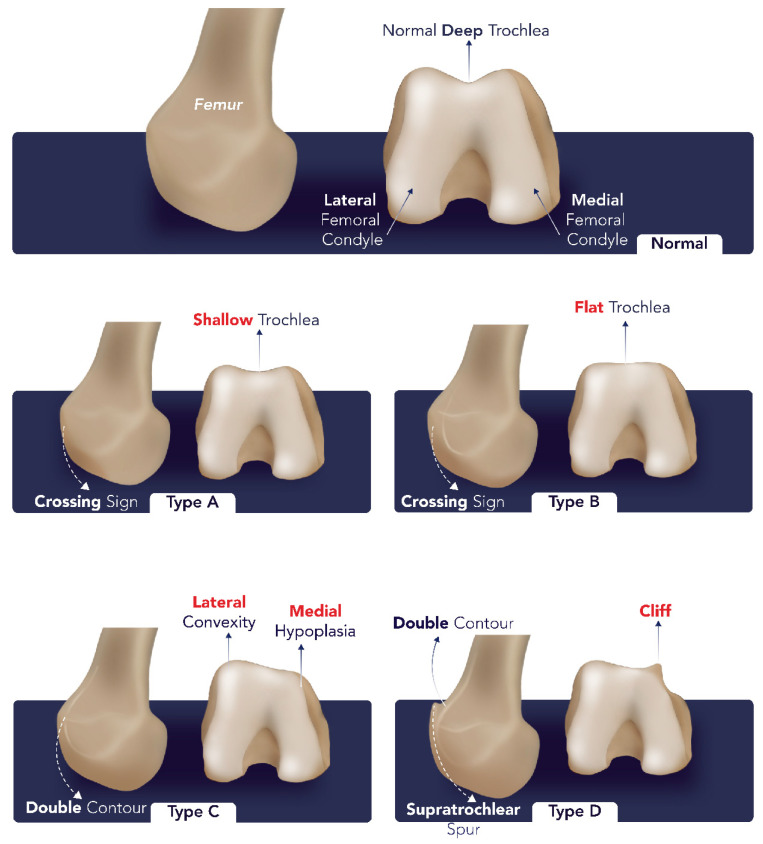
Classification of trochlear dysplasia. Type A: A shallow trochlear groove with mild dysplasia, but no supratrochlear spur. Type B: A flat or convex trochlear groove indicating more pronounced dysplasia with a prominent supratrochlear spur. Type C: A lateral trochlear facet that is hypoplastic or underdeveloped, leading to severe asymmetry in the trochlear groove. Type D: A “cliff” pattern in the trochlear groove with a sharp transition, marked by a large supratrochlear spur and asymmetry, often associated with the highest risk of patellar instability. The image was obtained from the University of Washington Medical Imaging database with the required permissions.

Physical examination may reveal episodes of knee instability or “giving way”, positive apprehension test, crepitation, and discomfort. Accurate diagnosis necessitates a detailed medical history, a thorough physical examination, and the utilization of appropriate imaging modalities. Radiographs can reveal various morphological features related to patellar malalignment, including patella alta and baja, lateral patellar tilt, trochlear or patellar dysplasia, and lateralization of the tibial tuberosity [5,24,29,30]. Trochlear dysplasia, as classified by Dejour et al. [31,32], is categorized into four distinct types based on imaging characteristics (Table 1) (Figure 5).

While radiography provides useful initial information, CT scans show details that are particularly valuable for surgical planning due to their superiority in depicting cortical bone defects. MRIs, in contrast, provide more detailed anatomical information about the knee cartilage, tendons, ligaments, muscles, and other internal components [33,34]. Additionally, several measurements, such as the tibial tuberosity–trochlear groove (TT-TG) distance and the tibial tuberosity–posterior cruciate ligament(TT-PCL), have been proposed as valuable tools for evaluating patellofemoral instability by assessing the alignment of the patellar tendon relative to the trochlea [35].

### 3.3. Osgood–Schlatter Disease and Sinding–Larsen–Johansson Disease

The Osgood–Schlatter disease (OSD) and Sinding–Larsen–Johansson disease (SLJD) are two self-limiting patellar disorders that commonly occur during growth spurts at the ages of 10–15 years. Athletes engaging in activities such as sprinting, hiking, basketball, and volleyball are at an increased risk. They usually present with anterior knee pain [36,37].

Diagnosing SLJD using radiographs can be challenging. As the condition progresses, radiographs may show dystrophic calcification and/or ossification adjacent to the inferior pole of the patella. Ultrasound imaging provides additional information by revealing specific areas of hypoechogenicity, increased thickness, and heterogeneity in the proximal patellar tendon, particularly in the posterior fibers attached to the patella. MRI also shows an increased signal intensity on T2/STIR seen at the inferior pole of the patella and proximal patellar tendon [38,39,40].

To diagnose OSD, it is essential to correlate the patient’s symptoms with imaging findings. Patients typically experience gradual pain localized to the tibial tubercle, which worsens with activity and improves with rest, often without a history of trauma. Initial radiography in the acute phase shows the loss of distinct boundaries of the patellar tendon and increased prominence of the surrounding soft tissues. Bone fragmentation at the tibial tuberosity usually becomes apparent after three to four weeks of symptoms.

**Figure 6:**
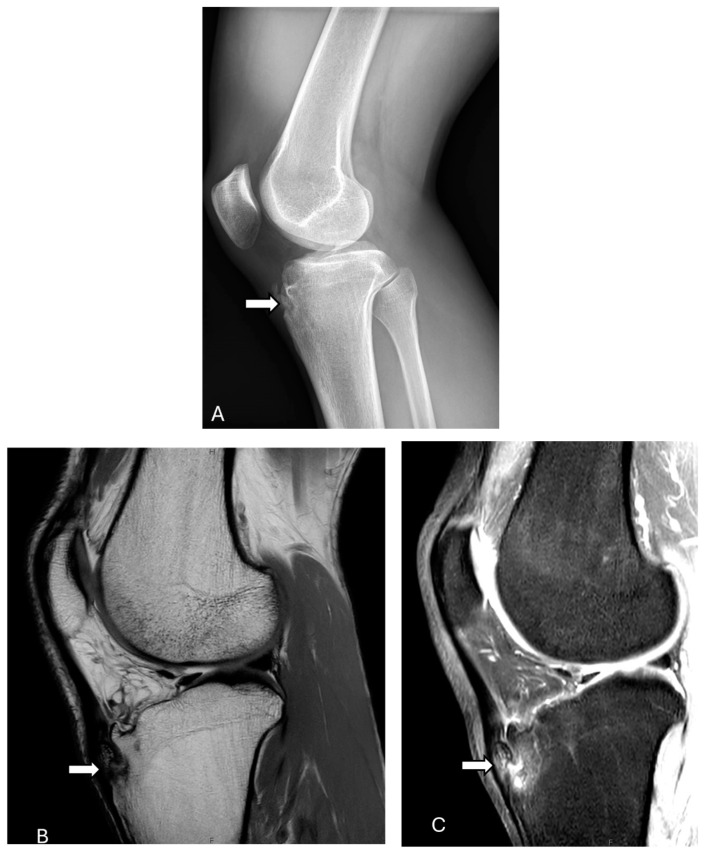
Osgood–Schlatter disease. Lateral knee X-ray (**A**), sagittal T1-weighted (**B**), and PD fat-suppressed (**C**) images show well-corticated ossific densities adjacent to the tibial tuberosity (arrows). These ossifications follow the signal intensity of bone on MR images, likely sequela of Osgood–Schlatter disease. The images were obtained from the University of Washington Medical Imaging database with the required permissions.

Sonographic findings of OSD include swelling of the soft tissues adjacent to the tibial tubercle, fragmentation, and irregularity of the ossification center with decreased internal echogenicity, thickening of the distal patellar tendon, and infrapatellar bursitis. On MRI, it is characterized by soft-tissue swelling anterior to the tibial tuberosity, loss of the sharp inferior angle of the infrapatellar fat pad (Hoffa’s fat pad), thickening and swelling of the distal patellar tendon, enlargement of the deep infrapatellar bursa, and evidence of bone marrow edema at the tibial tuberosity [37,41,42,43,44] (Figure 6).

### 3.4. Osteochondral Injury

Common causes of osteochondral injury in the knee include compaction, shearing, and avulsion. Acute injuries are frequently minor and may not result in significant functional impairment. Additionally, radiographs can appear normal even in the presence of an osteochondral lesion. Therefore, in cases of discomfort, effusion, and mechanical symptoms, additional diagnostic imaging such as MRI or CT is recommended.

The imaging appearance of osteochondral injuries varies depending on the stage of the injury [45,46,47,48]. The Osteochondral Injury Staging System helps classify cartilage damage based on severity [49] (Table 2).

### 3.5. Excessive Lateral Pressure Syndrome

Excessive lateral pressure syndrome (ELPS) or lateral patellar compression syndrome (LCPS) happens when there is an unusual lateral shift of the patella without translation, subluxation, or dislocation. It results in uneven stress on the medial and lateral articular surfaces and can be caused by shortness of the lateral retinaculum, tightness of the vastus lateralis, laxity of the medial retinaculum, or weakness of the vastus medialis [50,51,52,53]. Symptoms include pain in the inferomedial patella and anteromedial joint line during prolonged knee flexion or while ascending or descending stairs, as well as limitation of motion (LOM) in extension. Notably, the pain is usually persistent, with limited response to medication, physical therapy, or bracing [54].

Radiography is commonly used to diagnose LCPS. Patellar tilt is identified with traditional measures like the congruence angle (CA) and patellar tilting angle (PTA). The CA angle assesses the alignment of the patella within the trochlear groove, while the PTA measures the tilt of the patella relative to the femoral trochlea. Both angles are commonly used but have limitations in precision [51,55]. Yang et al. [51] proposed using LPCA obtained from a Merchant view radiograph with the knee in 30° flexion as an alternative technique. The LPCA considers the impact of osteophyte development, which can cause the lateral articular surface of the patella to become deeper and more curved than in healthy individuals, providing a potentially more reliable diagnostic measure (Figure 1). Additionally, MRI is considered the most advanced imaging method for more accurate diagnosis and monitoring, with a reported sensitivity of 70% and 90% for detecting LPCS. Common MRI findings include cartilage and subchondral edema, articular cartilage defects, tissue hypertrophy, and joint effusion [56,57,58].

### 3.6. Patellar Tendon Pathologies

#### 3.6.1. Patellar Tendon Tear

A ruptured patellar tendon represents a significant injury to the knee’s extensor mechanism. This injury typically results from trauma to the insertion of the patellar tendon at the tibia or the lower pole of the patella. It most commonly affects physically active males in their third or fourth decade of life but can also occur in older adults with an underlying systemic illness such as chronic renal disease, rheumatoid arthritis, diabetes mellitus, or systemic lupus erythematosus. Acute tendon tears are generally repairable, whereas chronic ones need tendon reconstruction [59,60,61,62].

**Figure 7:**
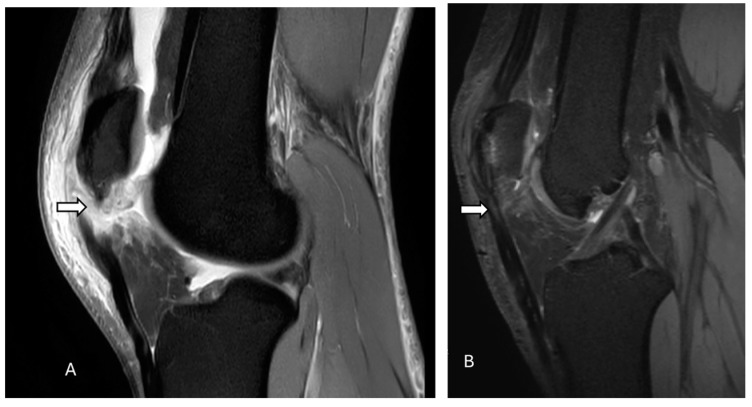
Complete tear of the patellar tendon. Proton density fat-suppressed sagittal images show a complete tear of the proximal patellar tendon (arrow) before (**A**) and after surgery (**B**) near its attachment to the patella, with approximately 10 mm retraction of the distal tendon stump. There is marked surrounding soft tissue edema. Mild laxity of the intact quadriceps tendon is evident. The postoperative image (**B**) demonstrates an intact repaired patellar tendon with the expected postsurgical signal intensity (arrow) without any focal tear. The images were obtained from the University of Washington Medical Imaging database with the required permissions.

**Figure 8:**
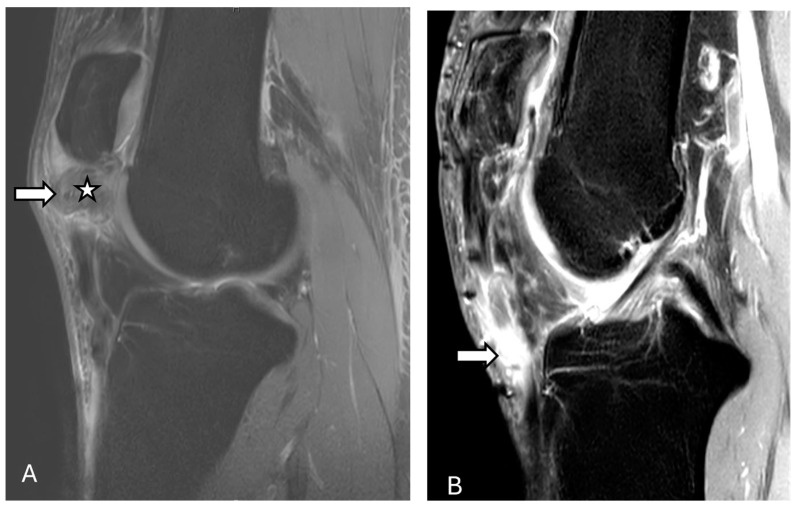
Patella alta with complete tear of the patellar tendon. Proton density fat-suppressed sagittal images before (**A**) and after (**B**) surgery reveal patella alta along with complete tear of the patellar tendon at its proximal attachment (arrow), with a 21 mm tendon gap and hematoma across the tendon gap (star). Intrasubstance fluid signal is present within the proximal tendon stump, which is heterogeneous and thickened. Furthermore, there is mild edema within the attached distal tendon, extending to the tibial tuberosity. Post-operative images (**B**) show thickening, irregularity, and increased intrasubstance signal within the distal patellar tendon (arrow), concerning failure of patellar tendon reconstruction. The images were obtained from the University of Washington Medical Imaging database with the required permissions.

On radiographs, the normal patellar tendon appears as a homogenous, band-like soft tissue density, with fat visible on both sides. A ruptured patellar tending on MRI is indicated by a defect that appears with homogeneous, low signal intensity on T1-weighted images and a bright fluid-filled cleft on fluid-sensitive sequences (Figure 7 and Figure 8). On ultrasonography, there is a disruption of the normal fibrillar texture of the tendon along its course seen as fluid-filled gaps (anechoic clefts) or areas of unevenly increased echogenicity (heterogeneously hyperechoic material), often due to fat from Hoffa’s fat pad infiltrating the tear [63].

#### 3.6.2. Patellar Tendinitis/Tendinosis

**Figure 9:**
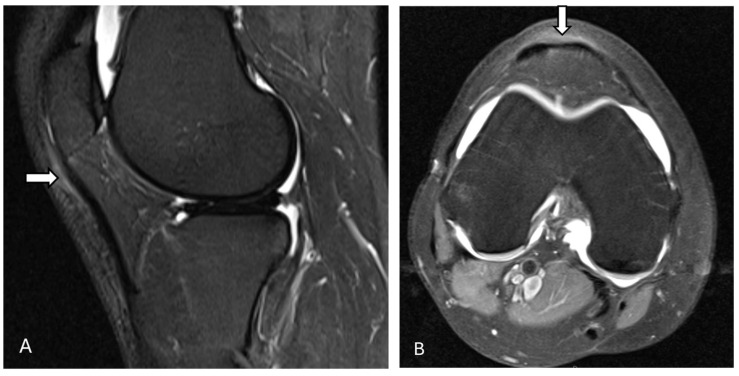
Patellar tendinosis. Proton density fat-suppressed images (**A**,**B**) show increased signal intensity of the patellar tendon at its patellar attachment (arrow), without focal tear consistent with moderate tendinosis of the proximal patellar tendon. The images were obtained from the University of Washington Medical Imaging database with the required permissions.

Patellar tendinopathy commonly affects physically active individuals engaging in sports involving running and jumping. It is primarily characterized by tendinosis, which involves degeneration of the tendon, leading to pain at the inferior pole of the patella, which is exacerbated by knee extension [64,65,66,67]. While clinical assessment is generally reliable for diagnosing patellar tendinopathy, confirmatory diagnostic imaging exists. Ultrasound imaging may reveal abnormal thickening of the patellar tendon and an increased cross-sectional area. MRI results (Figure 9) indicate increased T2 signal intensity secondary to degenerative changes; increased tendon thickness; and, in advanced cases, calcification, which appears as low signal intensity [68,69,70,71].

### 3.7. Chondromalacia Patella

The term “chondromalacia” derives from Greek, where “chondros” means cartilage and “malakia” signifies softening. It involves the degeneration of the articular hyaline cartilage of the patella, which can result from post-traumatic injuries, repetitive minor injuries, or medical injections [72,73]. Chondromalacia patella is commonly observed in runners and climbers, particularly young females. Furthermore, symptoms typically include anterior knee pain while descending stairs, while kneeling, and after prolonged sitting [72,74,75].

**Figure 10:**
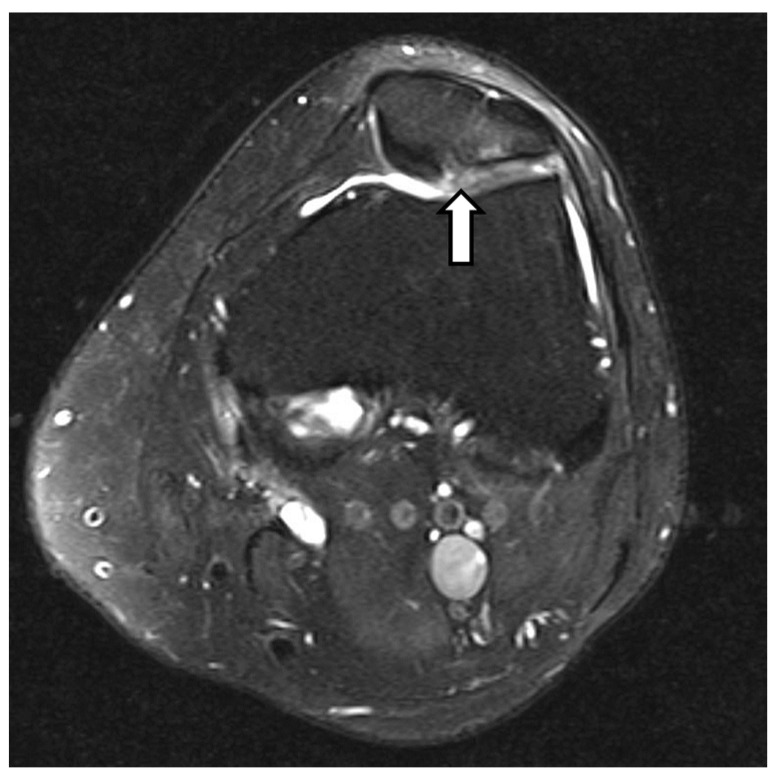
Chondromalacia of patellar cartilage. Axial proton density fat-suppressed image demonstrates multifocal high-grade fissuring (arrow) and heterogeneity throughout the lateral facet and median ridge patellar cartilage with significant cartilage thinning. The image was obtained from the University of Washington Medical Imaging database with the required permissions.

Radiographs have limited sensitivity and specificity during the initial stages of chondromalacia patella. However, at more advanced stages, they may show cystic alterations or a reduction in joint space. Radiography could be used to assess underlying conditions such as patella alta, patella baja, or trochlear dysplasia, as well as to identify secondary patellofemoral osteoarthritis [72]. CT arthrography may be unrevealing in early chondral injuries but provides useful information on torsional anomalies and patellofemoral alignment. For the most accurate assessment of articular cartilage, fluid-sensitive, fat-saturated MRI sequences, such as proton density imaging (Figure 10), are preferred. These imaging techniques typically highlight abnormal cartilage with increased signal intensity compared to normal cartilage [72,76,77].

### 3.8. Prepatellar Bursitis

#### 3.8.1. Anatomy Prepatellar Bursae

**Figure 11:**
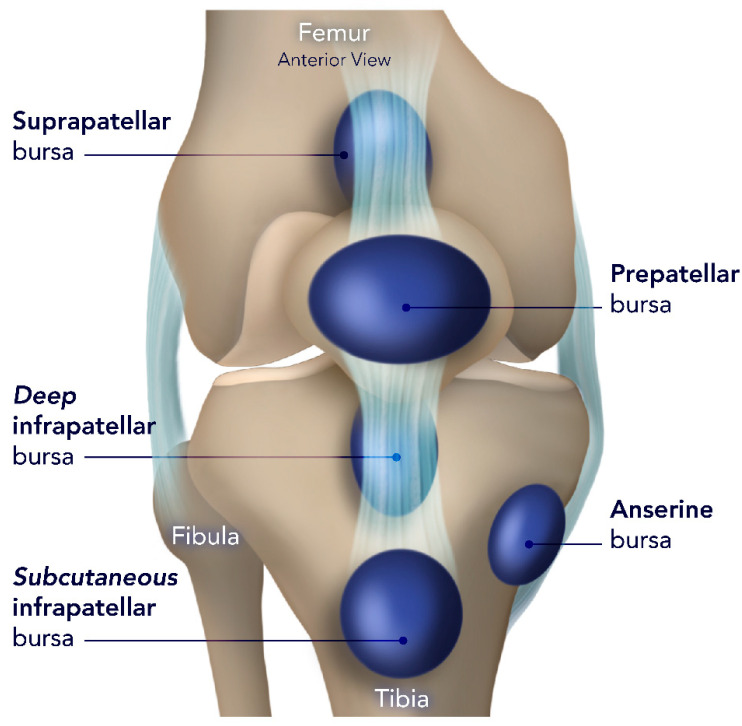
Schematic representation of the knee bursae, illustrating the anatomical locations and relationships of major bursae around the knee joint. Key bursae include (1) prepatellar bursa (located anterior to the patella), which protects the knee from friction; (2) infrapatellar bursa (divided into superficial and deep components), which cushions the patellar tendon; (3) suprapatellar bursa (located above the patella), facilitating movement between the quadriceps tendon and femur; and (4) pes anserinus bursa (medial aspect of the knee), reducing friction between the tendons of the sartorius, gracilis, and semitendinosus muscles and the tibia. The image was obtained from the University of Washington Medical Imaging database with the required permissions.

The patella is encircled by four bursae (Figure 11) that act as cushions between different structures. Inflammation, infection, hemorrhage, and chronic and acute trauma can result in bursitis. Common inflammatory causes include gout and rheumatoid arthritis [78,79,80,81].

Bursitis often results in localized discomfort and swelling in acute phases, while chronic conditions are usually painless. Multiple risk factors are associated with the development of prepatellar or superficial infrapatellar bursitis, including chronic glucocorticoid use and old age, both of which contribute to thinning of the skin over these bursae and the limited nerve supply to the skin in these areas. Additionally, the presence of tophi and rheumatoid nodules at the pressure points contributes to the development of this condition [78,79].

Acute prepatellar bursitis and superficial infrapatellar bursitis are typically caused by infection from common bacteria found on the skin and, less commonly, by gout or trauma (hemobursa). Typically, they exhibit localized redness, swelling, and significant discomfort in front of the patella or patellar tendon. Approximately 30 percent of individuals may exhibit fever and knee effusion, and in cases where the bursa ruptures, rapidly extending cellulitis to the sides of the knee and along the leg may develop [82,83,84,85].

Chronic prepatellar bursitis and superficial infrapatellar bursitis typically manifest as a painless, rounded swelling filled with fluid located in front of the patella or the patellar tendon. The lump may feel cool or slightly warm to the touch. Typically, there is a past occurrence of trauma or chronic usage, and the presence of a nodular surface indicates the possibility of tophaceous gout or nodular rheumatoid arthritis [86,87].

The first step in assessing knee bursitis is ruling out septic bursitis. This involves performing bursal aspirations and analyzing it for white cell count and differential, glucose content, crystal identification, Gram stain, and culture. Imaging modalities could be used when there are symptoms of local or systemic inflammation or suspicion of gout or septic bursitis. Ultrasound-guided aspiration may be employed when a physical examination fails to definitively detect the presence of an effusion. MRI is rarely needed for diagnostic purposes; however, it may be utilized if there is a suspicion of underlying malignancies or osteomyelitis [78,79,81,87,88].

#### 3.8.2. Deep Infrapatellar Bursae

The deep infrapatellar bursa (DIB) is located between the patellar tendon and the upper front part of the tibia. It can be observed on lateral paramedian sagittal MR imaging and has average dimensions of 2.1–2.7 mm from front to back and 7.3–9.1 mm from top to bottom [89,90]. 

**Figure 12:**
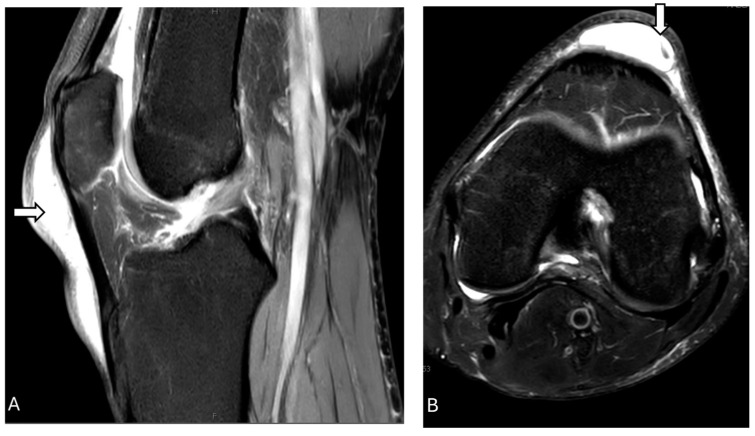
Prepatellar bursitis. Sagittal (**A**) and axial (**B**) proton density fat-suppressed images show moderate amounts of fluid within the prepatellar (arrow) and infrapatellar bursae appearing as hyper signal intensity anterior to the patella with surrounding edema, suggesting bursitis. The images were obtained from the University of Washington Medical Imaging database with the required permissions.

The differential diagnosis of bursitis includes OSD and SLJD. Both are conditions of excessive strain in adolescents participating in sports [91]. In SLJD, pain, tenderness, and swelling are experienced at the lower pole of the patella, while in OS, they occur at the tibial tuberosity. The best modality for knee bursae assessment is MRI, which can identify even a little effusion. MRI reveals bursitis as confined fluid signal intensity in the anticipated location (Figure 12). On gradient-echo (GRE) imaging, hemorrhagic collections appear as areas of signal drop due to susceptibility artifact [92,93]. Ultrasound is a useful tool for evaluating superficial bursitis in patients who are unable to have an MRI. It has a sensitivity of 86.67% and a specificity of 100% when compared to MRI. Draghi et al. discovered that ultrasound has less sensitivity compared to MRI when examining the suprapatellar bursa [94].

### 3.9. Osteomyletis of Patella

Osteomyelitis of the patella (OMP) is a rare disease that primarily affects children between the ages of 5 and 15 [95,96,97]. It presents with various symptoms and does not typically cause fever, which can make diagnosis challenging [96]. OMP is unlikely to occur before the age of 5 because the patella is mainly composed of cartilage at this age [98]. OMP typically manifests as a hematogenous disease with no physical injury, with staphylococcus aureus being the most encountered infecting microorganism [95,96,97,99,100]. Adults who are at higher risk of having primary hematogenous osteomyelitis frequently have a history of trauma, HIV infection, or intravenous drug use [95].

Clinical examination is the initial step in suspecting OMP, especially in individuals with anterior knee discomfort and point tenderness over the patella [95]. Laboratory testing frequently indicated normal white blood count, even in the presence of acute osteomyelitis. C-reactive protein is mostly raised and could be used to measure response to medication [95,101].

Radiography is the initial imaging modality most commonly employed and may be normal since periosteum forms early on the anterior patellar surface. However, the other patellar margins retain chondro-osseous interfaces that persist through adolescence, and evidence of OMP may be exhibited later. As a result, patellar infections may not exhibit the characteristic periosteal elevation seen in other bone localizations. However, baseline radiographs can rule out other possible diagnoses and lead to further investigations [4,100,102].

**Figure 13:**
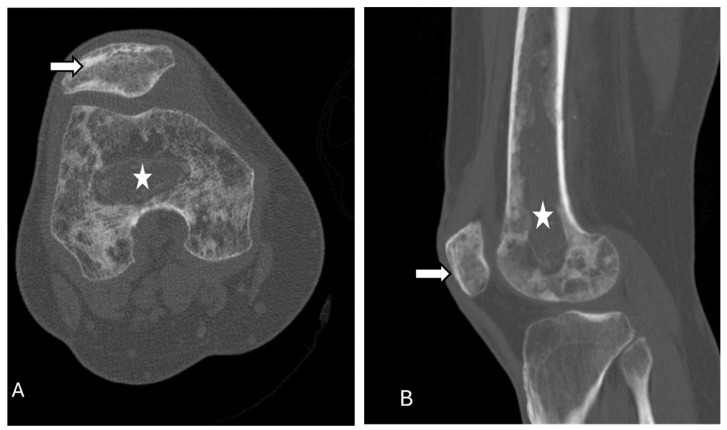
Osteomyelitis. Axial (**A**) and sagittal (**B**) CT scans of knee show diffusely osteopenic femur and patella with a moth-eaten appearance (arrows), likely secondary to osteomyelitis healing changes and disuse. There is also another area of focal osteopenia adjacent to the antibiotic beads (stars) within the femoral medullary cavity. The images were obtained from the University of Washington Medical Imaging database with the required permissions.

MRI is the gold standard for the diagnosis of OMP [96,99,100] (Figure 13). The appearance of cortical erosions, bone marrow edema, periostitis, soft tissue or bone abscesses, and possible joint effusion can be detected [103,104]. A normal MRI can almost rule out osteomyelitis. MRI and CT scans can be utilized to assess the extent of bone involvement and guide appropriate intervention [96,101].

### 3.10. Metabolic Diseases

Gout is inflammatory arthritis caused by the deposition of monosodium urate (MSU) crystals. The most common location affected by this disease is the first metatarsophalangeal in 57.4% of patients [105,106]. In the knee region, acute gout attacks result in sudden pain, swelling, and erythema [107,108].

Gout is formally diagnosed based on clinical symptoms, elevated plasma urate levels, and joint/tophus aspiration with microscopical verification of MSU crystals [109]. MSU depositions in patients can be detected by ultrasound and dual-energy CT (DECT) according to the 2015 ACR/EULAR gout categorization criteria [110].

**Figure 14:**
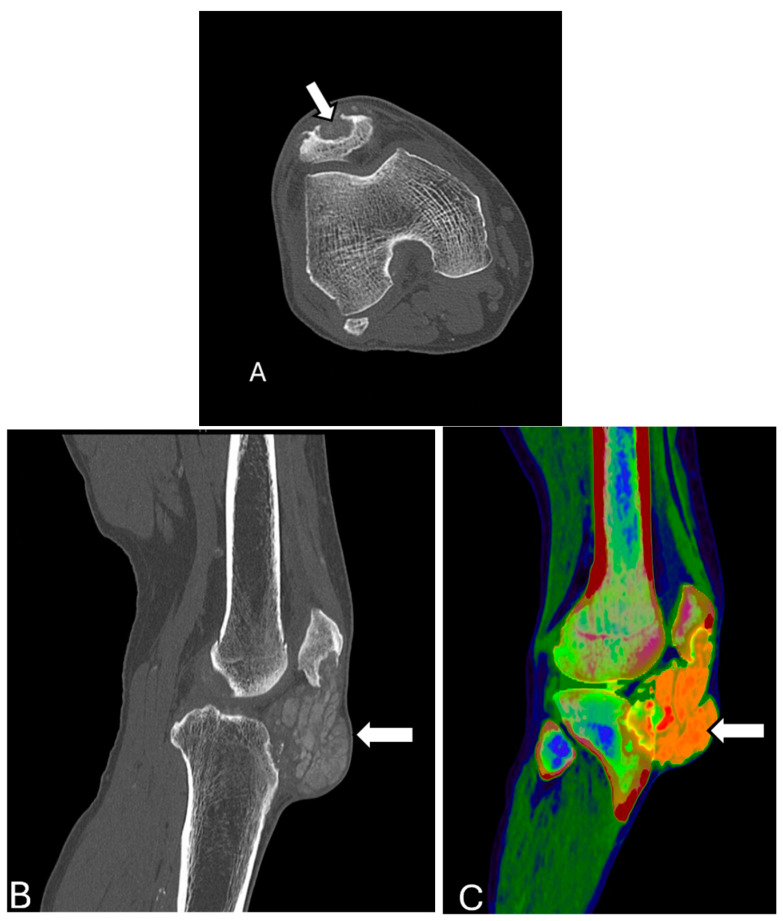
Gout. Axial (**A**) and sagittal (**B**) CT scan of the knee reveal a large patellar/prepatellar tophus (arrows) corresponding with urate crystal density on the color map (**C**). Multiple osseous erosions are seen within the tibial tubercle and the dorsal inferior patellar pole, representing gout arthropathy. The images were obtained from the University of Washington Medical Imaging database with the required permissions.

Radiographic features can be used to diagnose gout in advanced stages. Common radiographic signs of gout include juxta-articular erosions with a “punched-out” or “rat bite” appearance, characterized by sclerotic margins and overhanging edges (Figure 14). While bone mineralization is normal, pathologic fractures can arise due to weakened bones. In chronic gout, tophi are masses of focal soft-tissue mineralization near the joints. Knee tophi (Figure 14) are usually intra-articular and located in the infrapatellar fat pad, anterior joint recess, lateral femoral condyle rim, and intercondylar fossa, which could erode into adjacent bones, including the patella [111,112,113,114].

Ultrasonography can be used in diagnosis and monitoring therapy. The presence of a double contour sign on the surface of joint cartilage and a hyperechoic cloudy area surrounded by a hypoechoic border in joints or tendons are key diagnostic indicators of gout when using ultrasonography with a sensitivity of 83% and 65%, respectively. DECT is typically used to diagnose gout and can distinguish urate from calcium deposition [114,115,116]; however, a systematic review revealed that DECT could also be utilized to assess the response to medication, mortality, prognosis, and the likelihood of recurrent gout flares [117]. In a meta-analysis, DECT had polled sensitivity and specificity of 89% and 91%, respectively [118]. Nevertheless, there is a drawback to having false negative results, particularly in those who have experienced symptoms for less than six weeks [116].

MRI could identify gout arthropathy, gout tophi, synovial pannus, bone marrow edema, and soft-tissue edema in individuals with normal radiographs. On T1-weighted images, the tophi demonstrate consistent and uniform intermediate to low signal intensity, while on T2-weighted images, the signal intensity varies. The following gadolinium is administered, it might provide either homogeneous or peripheral enhancement. Although MRI abnormalities may be in favor of gout, they are not specific and require aspirate confirmation to differentiate from infections or malignancies [111,112,114,119,120].

### 3.11. Malignancies of Patella

**Figure 15:**
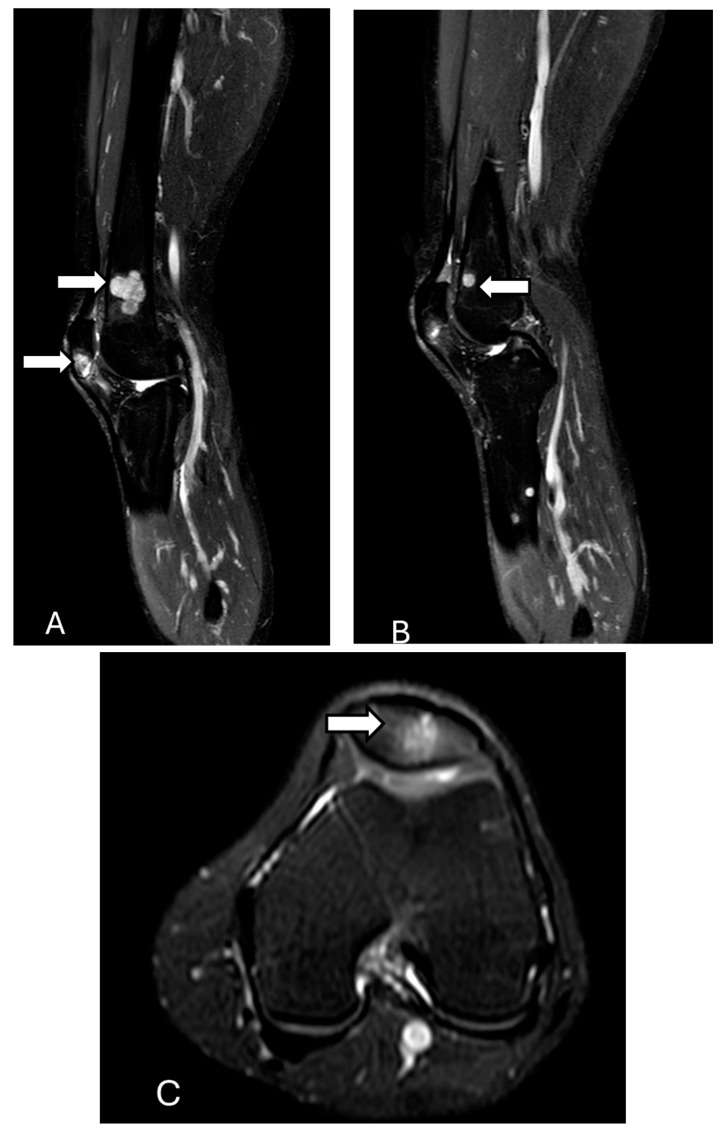
Epitheloid hemangioendothelioma. Sagittal (**A**,**B**) and axial (**C**) T1-weighted fat-suppressed post-contrast images show multiple foci of enhancement (arrows) throughout the distal femur, patella, and proximal tibia and fibula. These lesions are both medullary and cortically based with well-defined borders. Within the inferior pole of the patella, a similar lesion is observed. There is associated cortical thinning without evidence of cortical breakthrough. Findings are consistent with multiple metastasis with origin of epitheloid hemangioendothelioma. The images were obtained from the University of Washington Medical Imaging database with the required permissions.

Patellar tumors have a low incidence rate, accounting for only 0.1% of all primary bone tumors. Primary bone tumors can be either benign or malignant, while metastases can also occur. Patients can have symptoms such as anterior knee pain, the presence of a palpable mass, and knee swelling. The majority of patellar tumors are benign, accounting for around 80% of cases. The most prevalent types of benign patellar tumors are giant cell tumors (33%) and chondroblastoma (16%), followed by enchondroma, aneurysmal bone cysts, and osteoid osteoma. The primary malignant tumors in the patella are osteosarcoma (6%), hemangioendothelioma (3%) (Figure 15), malignant fibrous histiocytoma (1%), and angiosarcoma (less than 1%) [121,122,123,124,125]. In contrast to malignant tumors, which frequently have indistinct borders and no sclerotic rim, benign tumors frequently take the shape of geographic lesions with distinct edges and sclerotic rim. Furthermore, malignant patellar tumors are more likely to experience pathological fractures [126].

On radiographs, chondroblastoma and giant cell tumor (GCT) have an osteolytic (radiolucent) appearance. A thin sclerotic border is the distinguishing feature of chondroblastoma, in contrast to GCT. Chondroblastoma and GCT have been linked to secondary aneurysmal bone cyst formation, which contains hemorrhage/fluid material, giving it a fluid–fluid level appearance on fluid-sensitive MR images [126,127,128]. Chondroblastoma, unlike GCT, predominantly affects people under the age of twenty and shows prominent peri-lesional marrow edema [129].

Adenocarcinoma and squamous cell carcinoma of the lung are the most prevalent primary cancers that metastasize to the patella, although this is an uncommon occurrence. The radiographic characteristics of patellar metastases are specific to cancer type. Common observations include lytic lesions, pathological fractures, and periosteal reactions. Tissue sampling is the gold standard of diagnosis [125,130].

## 4. Future Directions in Imaging and Management

Recent advancements in imaging and diagnostic technologies have significantly enhanced the evaluation of patellar pathologies, providing earlier and more accurate detection. Advanced MRI techniques, such as T2 mapping and quantitative fat-suppression imaging, offer precise insights into the structural integrity of the patella and surrounding cartilage, enabling the identification of degeneration in its early stages [131,132,133]. Additionally, ultrasound elastography, including shear-wave elastography and compression elastography, further enhances the diagnostic accuracy of patellar tendinopathy by quantitatively assessing tissue stiffness. Unlike conventional ultrasound, which may show hypoechogenic regions in both symptomatic and asymptomatic tendons, USE provides more reliable information on tissue properties, helping to differentiate clinically significant tendinopathies from incidental findings [134,135,136,137]. Furthermore, the incorporation of artificial intelligence (AI) into radiologic assessments has the potential to automate image interpretation, reducing human error and improving diagnostic accuracy. These innovations have the potential to offer a more comprehensive approach to diagnosing patellar disorders, enabling clinicians to make better-informed decisions and develop tailored management strategies [138].

The management of patellar pathologies depends on the type of pathology, its severity, the patient’s age, and the level of physical activity. For many non-traumatic disorders, conservative treatment is the first-line approach and includes physiotherapy, strengthening exercises, and techniques such as immobilization and controlled weight-bearing [139]. Conditions that are best managed non-operatively include first-time patellar dislocations [19], OSD, SLJD, small and non-displaced osteochondral defects that do not hinder knee dynamics, lateral patellar compression syndrome, partial patellar tears with intact extensor mechanism, chondromalacia patella, and non-infected bursitis. The second treatment option for patellar pathology is minimally invasive arthroscopy, which is mostly utilized in the fixation and/or removal of osteochondral defects that derange knee dynamics [140]. Arthroscopy is also reserved for identifying the underlying cause of refractory anterior knee pain syndrome [141].

Furthermore, surgical intervention is reserved for more severe cases not responding to conservative management, patellar tumors, osteomyelitis of the patella, infected bursitis, recurrent patellar dislocation, and complete tears of the patellar tendon [142]. Future research should focus on integrating advanced imaging technologies into clinical practice to enhance diagnostic accuracy; standardize protocols; and enable earlier, more precise detection of patellar pathologies, ultimately improving patient outcomes through personalized care.

## 5. Conclusions

Patellar pathologies, particularly non-traumatic conditions, are among the leading causes of knee dysfunction, contributing to significant discomfort and functional limitations. Diagnosing these conditions can be challenging, as it requires a multifaceted approach that combines clinical evaluation; imaging; and, when necessary, histopathologic analysis. This study explores a broad range of non-traumatic patellar pathologies and their characteristic radiologic features, reviewing key imaging markers such as patellar tilt, the TT-TG distance, and CA. By integrating these findings with clinical assessments, clinicians can improve diagnostic accuracy and develop more effective, tailored treatment strategies. Early identification and intervention are key to preventing irreversible joint damage and improving long-term patient outcomes. As we move forward, advancing imaging technologies hold the potential to enhance early diagnosis and refine personalized treatment plans. Future research should focus on evaluating these emerging techniques to optimize patient care and further our understanding of patellar pathologies.

## Figures and Tables

**Table 1:** Trochlear dysplasia types.

Types	Characteristics
Type A	A fairly shallow trochlear structure: crossing sign, shallow trochlea, sulcus angle greater than 145° on axial views
Type B	Flat or convex trochlea: crossing sign along with a supratrochlear spur
Type C	Asymmetry in the trochlear facets with a hypoplastic medial condyle: crossing sign and a double contour sign
Type D	Combination of an asymmetrical trochlear facets and a vertical joint pattern with a cliff-like appearance: crossing sign, supratrochlear spur, and double contour sign

**Table 2:** Osteochondral Injury Staging System.

Stage	Description	Radiological Finding
Stage I	Injury limited to articular cartilage	Radiography: Normal MRI: Subchondral edema
Stage II	Cartilage injury with associated subchondral fracture but without detachment; thin sclerotic margin	MRI: Subchondral edema Radiography: Usually normal; may see fracture as sclerotic or osteopenic area MRI: Type A (acute)—edema CT: Cystic changes and/or edema Type B (chronic)—Non-displaced, incompletely undercut by fluid (MRI) or lucency (CT), open connection to articular cartilage
Stage III	Detached, non-displaced fragment	Radiography: Slight lucency between osteochondral fragments and remainder of the bone MRI: High signal around osteochondral fracture (rim sign), not displaced
Stage IV	Osteochondral fragment displaced, joint effusion present	Radiography: Increased lucency between osteochondral fragments and remainder of the bone, or loose body with donor site irregularity MRI: Joint effusion surrounding fragment and filling donor site
Stage V	Subchondral cyst formation with secondary degenerative changes	Radiography: Secondary osteoarthritis

## Data Availability

Not applicable.
